# Chronic intramammary infection by *Listeria monocytogenes* in a clinically healthy goat – a case report

**DOI:** 10.1186/s12917-019-1989-3

**Published:** 2019-07-05

**Authors:** Maria Filippa Addis, Tiziana Cubeddu, Ylenia Pilicchi, Stefano Rocca, Renata Piccinini

**Affiliations:** 1 Dipartimento di Medicina Veterinaria, Università degli Studi di Milano, Via G. Celoria 10, 20133 Milan, Italy; 2 Dipartimento di Medicina Veterinaria, Università degli Studi di Sassari, Via Vienna 2, 07100 Sassari, Italy

**Keywords:** *Listeria monocytogenes*, Goat, Milk, Intramammary infection, Subclinical mastitis, Bacterial shedding, Histopathology, Immunofluorescence, Human health, Foodborne disease

## Abstract

**Background:**

*Listeria monocytogenes* is a ubiquitous Gram-positive bacterium responsible for a severe foodborne disease in humans, and contaminated dairy products can be an important source of infection. Typically, infected dairy ruminants show clinical manifestations including encephalitis, septicemia, abortion, and diarrhea, but may also become asymptomatic carriers and shed *L. monocytogenes* in the feces acting as an important source of viable bacteria. Isolation from individual goat milk has been documented very rarely, and chronic, asymptomatic intramammary infection by *L. monocytogenes* with continuous milk shedding of viable bacteria has never been described in this dairy species.

**Case presentation:**

At the routine controls, cheese and bulk milk were positive for *L. monocytogenes* in a herd of 200 lactating Alpine goats, but none showed clinical signs of listeriosis. Individual milk was subjected to bacterial culture and a clinically healthy goat was identified as affected by a chronic intramammary infection (IMI) by *L. monocytogenes*. The goat had never shown clinical signs of mastitis or other diseases. Her right half-udder milk was positive to *L. monocytogenes* in two consecutive samples collected one week apart, as demonstrated by bacterial culture and molecular analysis. Mammary tissues collected after culling were also positive to *L. monocytogenes* by culture. Histological examination highlighted a chronic interstitial mastitis with leukocyte infiltration, atrophy of the alveoli and presence of *corpora amylacea*. Immunohistochemistry (IHC) and immunofluorescence (IF) confirmed the presence of high numbers of bacteria in the lumen of mammary alveoli, with intracellular bacteria mainly located in macrophages, but also present in neutrophils and epithelial cells. After culling of the positive goat, bulk tank milk tested negative to *L. monocytogenes* at the following controls.

**Conclusion:**

This study demonstrates that *L. monocytogenes* can establish a chronic, subclinical IMI in goats with high numbers of bacteria shed in milk, representing a source of contamination for the herd and its dairy products. This underscores the importance of frequently monitoring all dairy herds that sell directly milk and/or fresh cheese and indicates that a chronic *L. monocytogenes* IMI should also be considered as source of bacteria when bulk tank milk contamination is detected in a dairy goat farm.

## Background

*Listeria monocytogenes* is a well-known pathogen affecting both humans and animals. It is considered a ubiquitous microorganism able to survive in many different environments (surface water, soil, sewage, plant material etc.) and in adverse conditions, such as high salt concentration, low temperature (growth already at 0.4 °C), and high temperature (maximum 45 °C), over a broad spectrum of pH and in a low water activity [1]. The pathogenic potential of *L. monocytogenes* is represented by its being an intracellular pathogen able to penetrate different host cells, thus affecting a wide range of animals. *L. monocytogenes* is considered the most pathogenic species for small ruminants [2]. Listeriosis can appear in three forms: encephalitis, septicemia, and intrauterine infections (which may lead to abortion). Less common outcomes are mastitis, iritis and keratoconjunctivitis. In addition to clinically evident forms, the animals may be asymptomatic carriers, shedding the bacterium in the feces and contaminating the environment [1, 2]. In the dairy herd, pathogen transmission can occur through the ingestion of contaminated water or food. In the milking parlor, the microorganism can contaminate the milk as a consequence of poor hygiene. In 2017, the European Food Safety Authority reported the occurrence of *L. monocytogenes* in 2.4% of goat, sheep and cow soft and semi-soft cheeses made from raw or low-heat-treated milk. The European Authority also claimed a significantly increasing trend of confirmed human listeriosis cases in the years 2013–17 in the EU/EEA, caused by the ingestion of different foods [3]. Therefore, this bacterium still represents a public health hazard for the ability of some strains to resist standard pasteurization conditions [4], but mainly for the manufacture of traditional cheeses using raw milk [5]. A further problem in controlling *L. monocytogenes* contamination in the farm is due to its capability to form biofilms [6]. Presence of bacteria in the milk could lead to adhesion to the milk-line with biofilm formation, mostly in the angle pipes, where washing is not optimal. In such situation, the microorganism could persist in the milking plant and contaminate the milk also after culling infected animals.

Subclinical and chronic mastitis without clinical signs of infection have been described in ewes and cows [7], and were likely responsible for bulk tank milk contamination [8–10]. Nonetheless, in reports concerning goat bulk tank milk contamination, source animals with intramammary infection (IMI) could not be detected [11]. Only one paper reported the isolation of *L. monocytogenes* from composite milk samples of two goats in Egypt [6], but in this case sampling was not repeated and therefore the presence of *L. monocytogenes* in milk as a result of a chronic IMI was not demonstrated.

Here, we describe a case of asymptomatic, chronic IMI by *L. monocytogenes* in a dairy goat and describe pathological features and microbial localization in the mammary tissue. Our data confirm that chronic IMI with bacterial shedding can be a likely source of contamination when positivity to *L. monocytogenes* is detected in goat bulk tank milk or dairy products.

## Herd description and case presentation

The herd consisted of 200 lactating Alpine goats housed in a free stall and milked using a machine equipped with automatic take-off device. The farmer produces cheese that is sold directly in the farm. Therefore, bacteriological analysis for foodborne pathogens is compulsory three times a year (February, June and October) and is carried out by the Regional Breeders Association of Lombardy (Associazione Regionale Allevatori della Lombardia, ARAL). At the control in June, cheese and bulk milk tested positive for *L. monocytogenes*, but no goat showed clinical signs of listeriosis. Therefore, all animals were screened by testing pools of 20 animals each; then, goats in the positive pool were sampled individually by collecting half-udder milk, and one goat shedding high numbers of viable *L. monocytogenes* in milk was eventually identified. The milk was sent to our laboratory for a confirmative diagnosis. The goat was clinically healthy and had never shown clinical signs of listeriosis or mastitis. The goat was culled on the following week. Thereafter, bulk milk tested repeatedly negative for *L. monocytogenes*.

## Sample collection and bacteriological analysis of milk and tissues

Half-udder milk collected for the confirmative diagnosis and on the day of culling was subjected to the somatic cell count (SCC) with a Bentley Somacount (Bentley, USA). The cytometric method of measuring SCC has been reported as reliable also in goat, even if it is difficult to interpret the results [12]. Milk SCC was very high in both udder halves at both samplings, collected one week apart: 5,141,000 cells/mL in the left half-udder and 6,429,000 cells/mL in the right half-udder on the first testing, 4,596,000 cells/mL and 5,714,000 cells/mL, respectively, on the day of culling.

At the slaughterhouse, the udder was retrieved and immediately transported to the laboratory in refrigerated conditions, where tissue samples were collected from cistern, proximal and distal parenchyma for bacteriological analysis and histological examination. Bacterial culture of milk and tissue samples was carried out in Aloa agar (Biolife, Italy) for the isolation of *Listeria* spp. and on blood agar (Oxoid, Italy) for the identification of other potential mammary pathogens. Plates were incubated at 37 °C for 24–48 h. Colonies with the typical morphological characteristics of *L. monocytogenes* were observed in Aloa agar (green-blue colonies surrounded by an opaque ring). Colony growth was observed in both right half-udder milk samples collected one week apart, as well as in the right half-udder proximal and distal parenchyma and cistern tissue. Bacterial load in milk was > 2,000 colony-forming units/mL in both right half-udder milk samples and ranged from 15 to 18 colonies in 1 cm^2^ of right half-udder tissue. Colonies were re-isolated for biochemical and molecular testing. Presumptive identification was confirmed at the genus level by API ID32 Strep (Biomerieux, France).

## Molecular identification of *L. monocytogenes*

Molecular identification was performed using 3 sets of species-specific primers on the case isolate as well as on the reference *L. monocytogenes* strain ATCC 19115 and on a characterized *L. ivanovii* strain as controls, as described by Tao and coworkers [13] with minor modifications. Amplification conditions were modified to further increase stringency by raising annealing temperature to 61 °C for primer sets Lm13 and Lm20, and to 65 °C for Lm8. Amplicons of the expected sizes were obtained for all the genes tested on the case isolate and on the *L. monocytogenes* reference strain while no amplification was obtained for *L. ivanovii*, confirming the isolate identification as *L. monocytogenes*.

## Histopathological findings

**Fig. 1:**
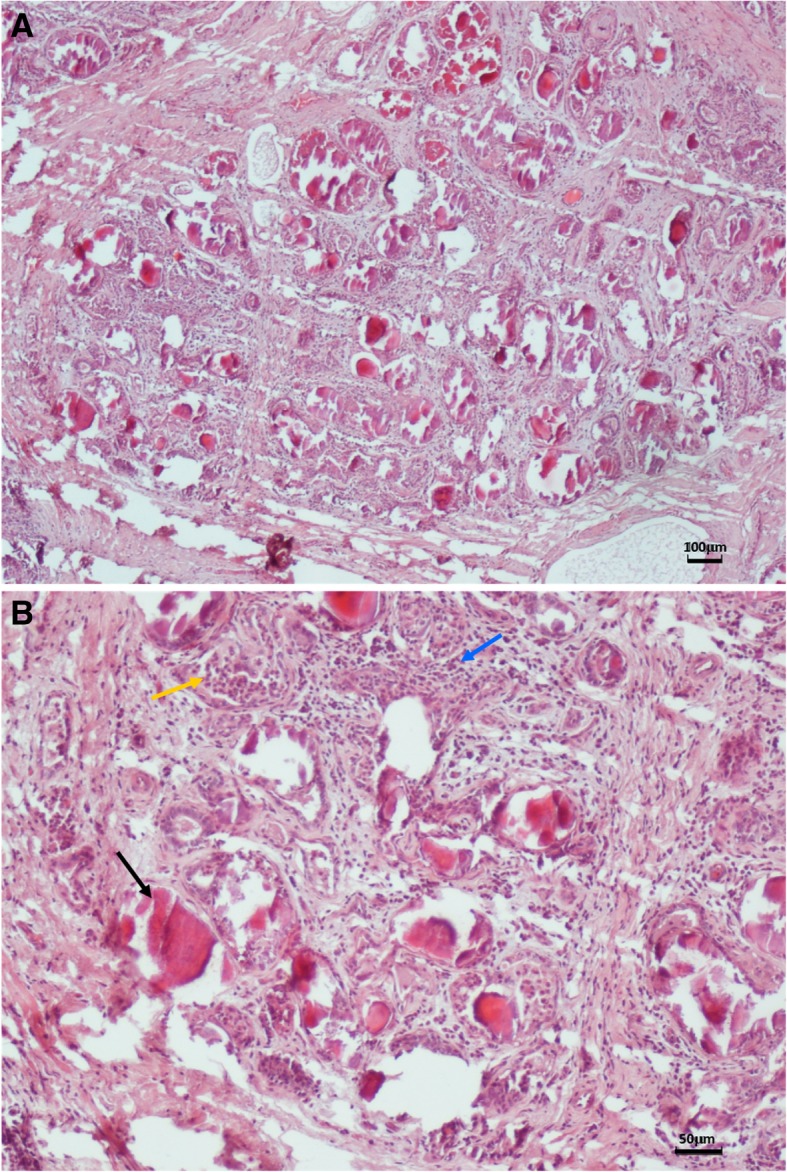
Hematoxylin-eosin stain of mammary tissue. **a** Optical microscopy image showing presence of interstitial mastitis. **b** Detail at higher enlargement showing alveolar atrophy and presence of *corpora amylacea* (black arrow), polymorphonuclear cells (yellow arrow) and inflammatory infiltration (blue arrow)

Fixation of mammary tissue samples and hematoxylin-eosin staining was carried out as described previously [14]. Histological examination of the right half-udder parenchyma highlighted an interstitial mastitis, with infiltration of macrophages and polymorphonuclear cells. In some areas of the tissue, atrophy of the alveoli and *corpora amylacea* could be observed (Fig. 1).

**Fig. 2:**
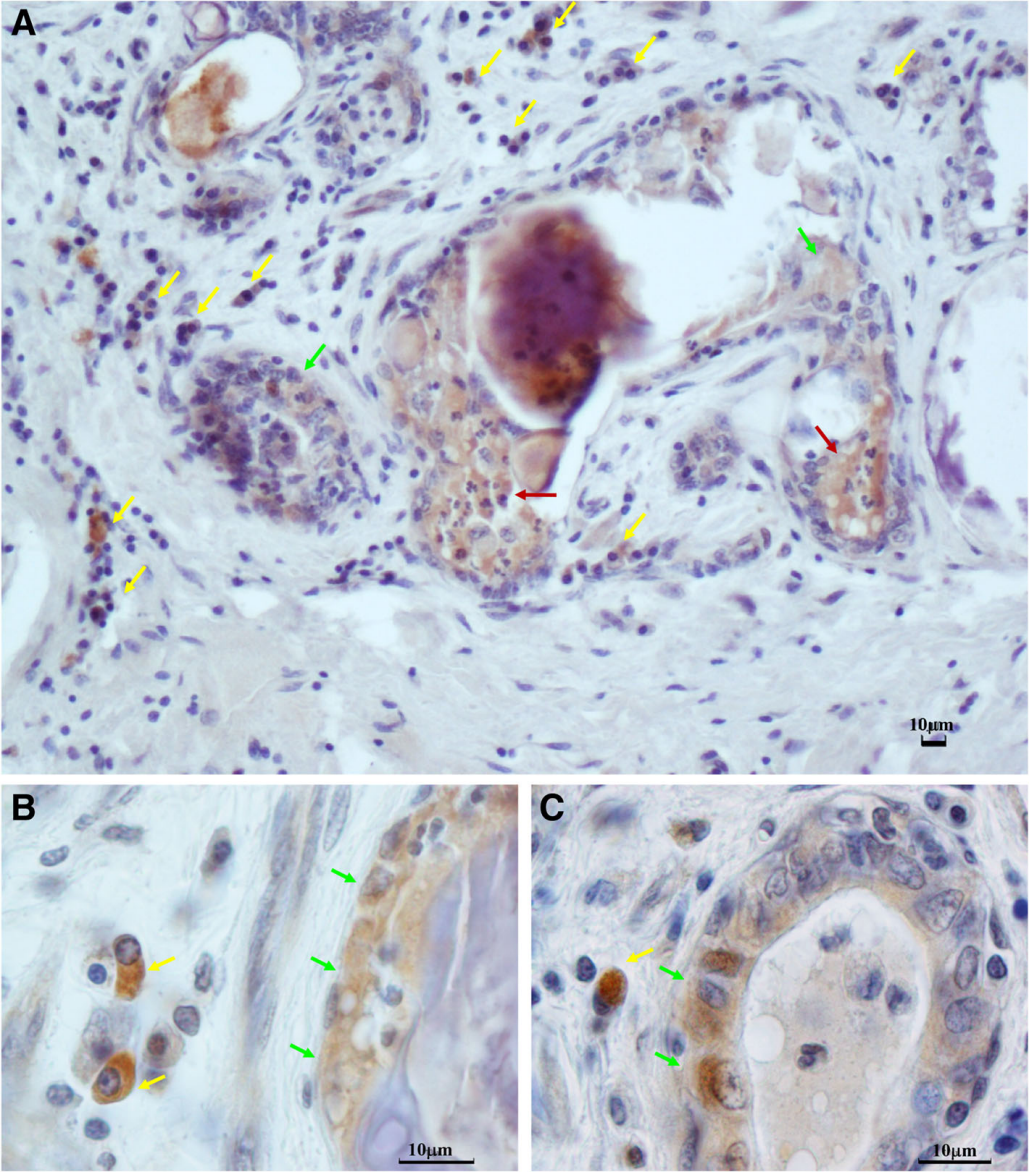
Immunohistochemistry detection of *L. monocytogenes* in mammary tissue. Nuclei are counterstained in blue with hematoxylin. **a** Positive reactions are indicated with arrows corresponding to different tissue locations: inside the mammary alveolus and in intra-alveolar neutrophils (red arrows); in tissue macrophages (yellow arrows), and in alveolar epithelial cells (green arrows. **b** Higher magnification showing a detail with positive macrophages (yellow arrows) and epithelial cells (green arrows). **c** positive signals in epithelial cells of a morphologically intact mammary alveolus (green arrows). A positive tissue macrophage can also be seen (yellow arrow)

Immunohistochemistry (IHC) was then carried out as described previously [15]. Bacteria were detected with a monoclonal antibody against *L. monocytogenes* LSH1 (Thermo Scientific), and nuclei were counterstained with hematoxylin. Numerous positive bacteria were observed within distal mammary tissues and inside the alveolar lumen (Fig. 2a). Positive cells were morphologically compatible with macrophages (Fig. 2, yellow arrows), epithelial cells (Fig. 2, green arrows) and polymorphonuclear cells (Fig. 2, red arrows).

## Immunofluorescence colocalization of *L. monocytogenes* with macrophages, neutrophils and epithelial cells

**Fig. 3:**
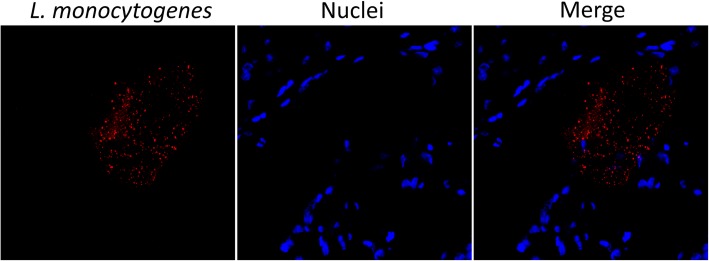
Immunofluorescence detection of *L. monocytogenes* in mammary tissue. Separate channel and overlay images are reported for bacteria (red) and nuclei (blue). The overlay image (merge) indicates that bacteria are free in the alveolar lumen

**Fig. 4:**
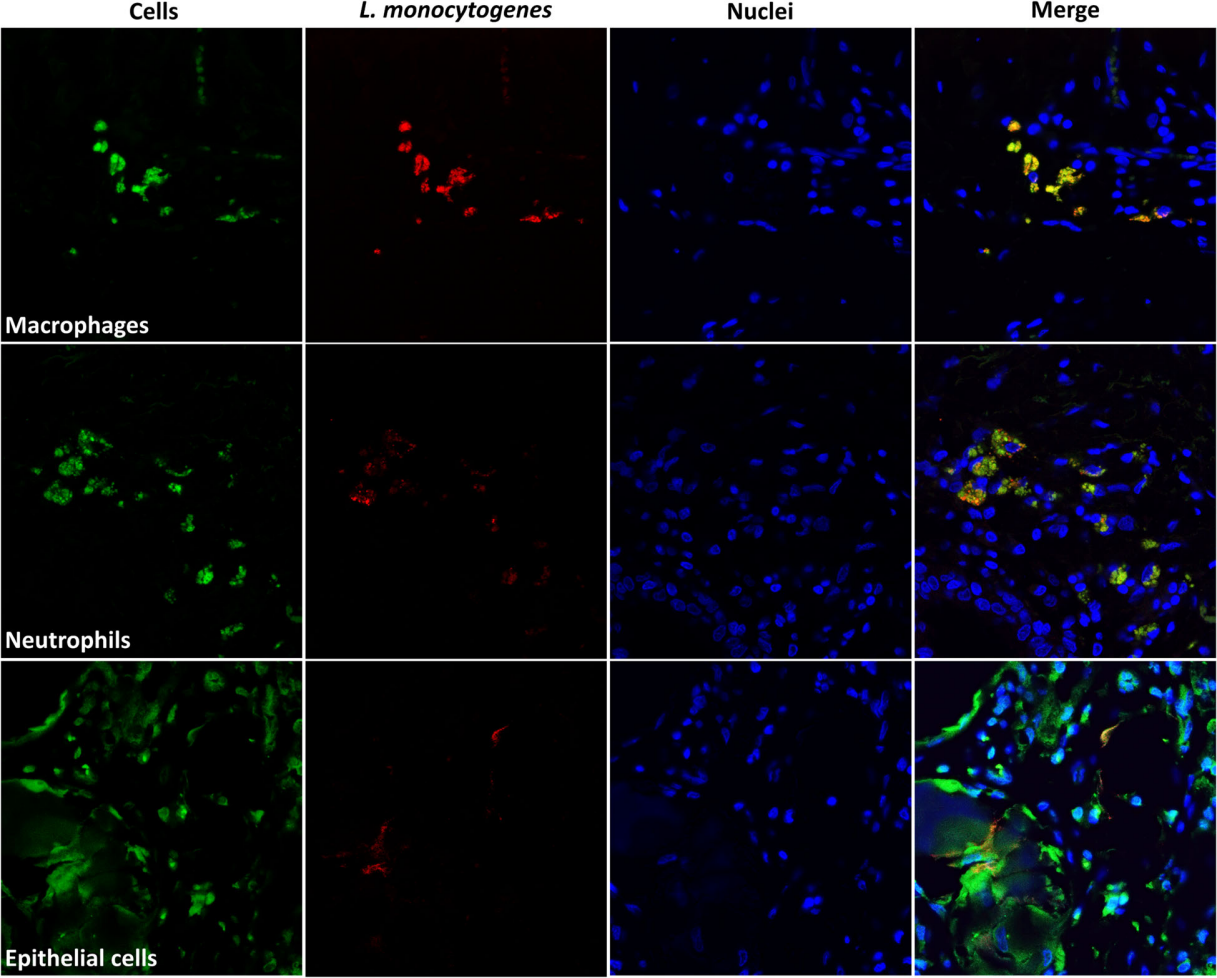
Immunofluorescence colocalization of *L. monocytogenes* and host cells. Separated channels with signals corresponding to the different cell types (green), *L. monocytogenes* (red), and nuclei (blue). The overlay image of all channels (merge) is reported on the right

To improve detection of bacterial cells within mammary tissues and infected cells, IF was also carried out as described previously [14]. The monoclonal antibodies LSH1, MAC 387 and Ly6B (clone 7/4) were used for detecting *L. monocytogenes*, macrophages and neutrophils, respectively. Antibody detection was done with rabbit anti-mouse secondary antibodies conjugated with Alexa-Fluor 555 for LSH1 and Alexa-Fluor 488 for MAC 387 and Ly6B. Anti-cytokeratin peptide 18 antibodies directly conjugated with fluorescein isothiocyanate (FITC) were used for epithelial cell detection. Nuclei were visualized by Hoechst staining. As a result, numerous *L. monocytogenes* organisms were detected within the alveolar lumen (Fig. 3). Upon colocalization with cellular markers to investigate intracellularly-located bacteria (Fig. 4), abundant *L. monocytogenes* signals (red) were observed mostly in macrophages (Fig. 4, top row) and neutrophils (Fig. 4, middle row). Although less abundant, bacterial signals were also present in correspondence of epithelial cells (Fig. 4, bottom row).

## Discussion and conclusions

While it has been reported that the goat can act as an asymptomatic carrier and shed *L. monocytogenes* in feces, persistent intramammary localization with repeated isolation of bacteria from milk has never been documented. This case report demonstrates that *L. monocytogenes* can invade the goat mammary tissue and establish a chronic IMI without showing any clinical sign, but resulting in the release of high numbers of viable bacteria in milk. Here, we followed the animal for one month observing that bacterial shedding was going on continuously. Following the first detection in bulk tank milk at the routine testing by the Regional Breeders Association and the identification of the infected goat, we repeatedly isolated the bacteria by culture and detected their presence within the mammary parenchyma. This demonstrated that presence of *L. monocytogenes* in milk was indeed due to an established intramammary colonization and not to sample contamination during milk collection or as a result of a transient presence of bacteria in the milk as a consequence of fecal excretion. The histopathological assessment revealed chronic, interstitial mastitis lesions compatible with a long-standing subclinical infection, especially indicated by the numerous *corpora amylacea* and leukocyte infiltrates scattered throughout the mammary tissue. The intracellular localization of bacteria, clearly observed by IHC and IF, further confirmed the establishment of a mammary tissue infection.

It has been demonstrated that intramammary inoculation of *L. monocytogenes* can lead to long-standing subclinical mastitis in sheep [16]. In that case, pathological features in experimentally infected animals were similar to those observed in subclinical mastitis caused by other intramammary pathogens. The lack of isolation of other, more frequent IMI agents in the present case report goat strongly indicates that *L. monocytogenes* was indeed the most likely cause of the observed mastitis.

It is worth mentioning that milk SCC is a less reliable indicator of inflammation than in other dairy animals [17, 18]. Therefore, the routine control of subclinical IMIs by SCC monitoring, such as with the California Mastitis Test, is less common than in cows or ewes. In this case, therefore, the high SCC values observed in both half udders would not provide a specific indication of an IMI or, even less, lead to the suspect of a listerial IMI.

In conclusion, this report highlights that controlling listeria colonization also in healthy animals is a crucial issue in goat farming. In fact, the establishment of a persistent, asymptomatic mastitis due to *L. monocytogenes* IMI has two main consequences: 1) the direct contamination of bulk milk for the shedding of high loads of *L. monocytogenes*; 2) the increase of environmental colonization and consequently the risk for contaminating milk and cheese during milk transformation procedures. This underscores the importance of carefully monitoring its presence in all the dairy herds that sell directly milk and/or fresh cheese for safeguarding public health, and that chronic IMI with bacterial shedding in milk should be considered when bulk tank milk contamination by *L. monocytogenes* is detected in a dairy goat farm.

## Data Availability

Not applicable.

## Abbreviations

ARAL: Associazione Regionale Allevatori della Lombardia
FITC: Fluorescein isothiocyanate
IF: Immunofluorescence
IHC: Immunohistochemistry
IMI: Intramammary infection
SCC: Somatic cell count
